# Targeting the Adenosine A2A Receptor as a Novel Therapeutic Approach for Renal Cell Carcinoma: Mechanisms and Clinical Trial Review

**DOI:** 10.3390/pharmaceutics16091127

**Published:** 2024-08-27

**Authors:** Ting-Yu Chen, Ya-Chuan Chang, Chia-Ying Yu, Wen-Wei Sung

**Affiliations:** 1 School of Medicine, Chung Shan Medical University, Taichung 40201, Taiwan; s1001144@gm.csmu.edu.tw (T.-Y.C.); raptor7037@gmail.com (Y.-C.C.); cyyu2015@gmail.com (C.-Y.Y.); 2 Department of Urology, Chung Shan Medical University Hospital, Taichung 40201, Taiwan; 3 Institute of Medicine, Chung Shan Medical University, Taichung 40201, Taiwan

**Keywords:** adenosine, renal cancer, metastasis, neoplasm, systemic therapy

## Abstract

Renal cell carcinoma (RCC) accounts for nearly 2% of cancers diagnosed worldwide. For metastatic RCC, targeted therapy is one of the most common treatment methods. It can include approaches that target vascular endothelial growth factor (VEGFR) or rely on immune checkpoint inhibitors or mTOR inhibitors. Adenosine A2A receptor (A2AR) is a type of widely distributed G-protein-coupled receptor (GPCR). Recently, an increasing number of studies suggest that the activation of A2AR can downregulate anti-tumor immune responses and prevent tumor growth. Currently, the data on A2AR antagonists in RCC treatment are still limited. Therefore, in this article, we further investigate the clinical trials investigating A2AR drugs in RCC. We also describe the epidemiology and current treatment of RCC, along with the physiological role of A2AR, and the types of A2AR drugs that are associated with tumor treatment.

## 1. Introduction

Renal cell carcinoma (RCC), which arises from the renal cortex or renal tubular epithelial cells, is the most common and most lethal type of urogenital cancer [1]. Currently, RCC makes up about 2% of cancers diagnosed worldwide, with its numbers still rising [1]. According to the ASCO guideline announced in 2022, the recommended treatments of metastatic RCC are immune checkpoint inhibitors (ICIs) or vascular endothelial growth factor receptor tyrosine kinase inhibitors (VEGFR TKIs). Combination therapies are often favored in poor-risk patients [1,2]. Despite the efficacy of targeted therapies, a high percentage of advanced RCC patients are resistant or refractory to current treatments. Therefore, new drugs targeting other oncogenic pathways are undoubtedly promising.

The adenosine A2A receptor (A2AR) is one novel drug target that has been drawing attention. Reportedly, the activation of the A2AR downstream pathway in tumor cells results in the downregulation of the immune system. Currently, increasing numbers of clinical trials are exploring the potential of A2AR antagonists as a combination treatment in targeted therapy [1].

Since the number of clinical trials studying A2AR drugs in RCC is still low, it might still be a while before we can conclude whether the A2AR drug is a reliable choice for RCC patients.

## 2. RCC Epidemiology

RCC accounts for more than 90% of kidney cancer (KC) cases. According to 2022 GLOBOCAN statistics, 434,840 new cases of kidney cancer were reported in 2022, and 155,953 new deaths were caused [3]. The prevalence of KC varies significantly worldwide [4], as the age-standardized rates (ASRs) in Europe and America are considerably higher than those elsewhere, with the highest ASR (12.2) reported in North America in 2020 [4]. Because the incidence rates are higher in developed countries, current estimates suggest that the rates in Africa and Asia will increase as populations shift to a Western lifestyle [5].

Over the past few decades, the rates of KC have been rising overall. From 1990 to 2016, the ASR experienced an increase from 4.48 to 4.97, a change of 10.94% [6]. The growing trend in the ASR is more significant in countries with higher socio-demographic indexes (SDIs) and greater life expectancy gains [6]. The age-standardized death rate (ASDR) showed a global increase from 1.93 to 2.00 from 1990 to 2016; however, the highest change in the ASDR was found in middle-SDI countries, which showed an increase of 38.46%. In contrast, in high-SDI countries, the ASDR decreased slightly by −1.14%. Better health care and disease prevention awareness in those countries might contribute to this downward trend [7]. The ASDR decreased the most significantly in regions with high kidney cancer burdens, including Southern Latin America and the Caribbean, which showed decreases of −16.94% and −31.75%, respectively [6].

The incidence of KC also varies significantly between sexes. According to GLOBOCAN data, in 2020, the ASR of KC in males was 6.1, while in females, it was 3.2 [3]. In Northern America, KC is estimated to be the fifth most common cancer (5%) diagnosed in men and the ninth most common (3%) in women [8]. Scientific evidence suggests that this difference is mainly derived from the differential expression of sex chromosome-linked genes in kidney tissue [9]. Family history plays an important role in about 3% of RCC cases, yet patients with inherited RCC do not necessarily have a family history. Among all the hereditary cancer syndromes observed in renal cell carcinoma, von Hippel–Lindau disease (VHL), a VHL gene mutation likely to result in clear cell RCC (ccRCC), is the most common [10]. Hereditary RCC syndromes are mainly autosomal dominant conditions [11]. Several RCC subtypes are related to inherited syndromes, with hereditary leiomyomatosis and RCC (HLRCC) and succinate dehydrogenase (SDH)-deficient RCC being most specifically associated, according to the 2016 WHO classification [11]. Furthermore, some RCC characteristics, such as diagnosis at a relatively young age (≤46 years) or the presence of bilateral or multifocal tumors [12], are potentially linked to a hereditary syndrome. The rates of RCC are higher in older populations, with a peak incidence between 60 and 70 years of age [12]. Lifestyle is also a key factor in the risk of RCC, with obesity, smoking, and excessive alcohol consumption all showing positive effects on RCC incidence [13].

Histologically, the three most common subtypes of RCC are clear cell RCC (70%), type I and II papillary RCC (10–15%), and chromophore RCC (5%). Among all subtypes, clear cell RCC is the most common and the most aggressive, with the highest tendency to metastasize to the liver, lungs, bones, and lymph nodes [1]. In the US, the overall 5-year survival rate in 2009–2015 for early-stage KC was a relatively high, at 93%, compared to the overall survival rate of 75% [14]. Therefore, early diagnosis of RCC can be life-saving. However, only about 10% of RCC patients are diagnosed with characteristic clinical symptoms. Up to 60% are incidentally diagnosed in routine ultrasound examinations [15].

### 2.1. Treatment of Localized RCC

For patients with stage I–III RCC, surgery is the preferred treatment. A T1 RCC tumor is less than 7 cm in size. The gold-standard treatment for T1 is partial nephrectomy (PN), which can preserve renal function and can be essential for patients with bilateral tumors or only one functioning kidney [16]. Clinical trials indicate that the effect of partial nephrectomy in T1 patients is no less than the effect of radical nephrectomy in terms of the OS, cancer-specific survival (CSS), or hazard ratio [17,18]. However, radical nephrectomy (RN) can serve as a substitute when partial nephrectomy is not possible. Active surveillance (AS) is another recommended treatment for T1 RCC patients, especially those with solid tumors of size <4 cm or Bosniak III/IV predominantly cystic lesions. People with these tumors have a relatively long natural history and a lower risk of metastasis; consequently, the risks posed by intervention and competing risks of death are likely to outweigh the benefits of treatment. AS should be highly personalized. It requires several measures, such as physical examination, laboratory evaluation, and abdominal and chest imaging. Renal mass biopsy (RMB) can be helpful in decision making regarding whether to initiate AS, but it poses a 5% risk of harmful complications [19,20].

Another emerging treatment for RCC of stages below T1b is stereotactic ablative body radiotherapy (SAbR). Although traditional fractioned radiotherapy was hindered by the relatively high radioresistance of RCC, SAbR may be able to overcome this through its higher dose-per-fraction and better precision to prevent progression and provide symptomatic relief. SAbR is minimally invasive because it does not require anesthesia or sedation. Thus, it is an option for elderly patients or high-risk surgical patients [21]. T2 RCC is characterized by solid tumors of size >7 cm without metastasis. For T2 patients, studies indicate a trend of lower recurrence-free survival (RFS) for PN than RN, but the OS of PN might be no less than that of RN [22,23]. Currently, the treatment of choice for T2 and T3 RCC is RN. In terms of the surgical method, laparoscopic RN is recommended for T2 tumors, while open surgery RN is often needed for T3 and T4 tumors [16].

### 2.2. Treatment of Metastatic RCC

Systemic treatments, surgery, and cytoreductive nephrectomy (CN) are the most useful methods for the treatment of metastatic RCC. Over the decades, systemic treatments for RCC have undergone a series of developments. Before the development of targeted therapy, IL-2 and interferon-α were the main treatments for metastatic RCC [24]. High doses of IL-2 can invigorate NK cells and CD4+ and CD8+ T cells, and IL-2 was one of the earliest cancer immunotherapies to be approved by the Food and Drug Administration (FDA) [24,25]. Studies show that IL-2 gives rise to long-lasting remission in up to 10% of patients, but the overwhelming adverse effects and fatality rate of high-dose IL-2 led this therapy to fall out of favor [26,27]. Currently, these cytokines commonly serve as adjuvant therapeutic options in RCC treatment protocols [28].

In the past 20 years, RCC treatments have evolved from nonspecific immune approaches to VEGF-targeted therapies combined with immune checkpoint inhibitors (ICIs) [29]. The overexpression of VEGF often results from an abnormality in the von Hippel–Lindau/hypoxia-inducible factor (VHL/HIF) pathway, which plays an important role in cancer development [30]. The activation of VEGF and its downstream pathway is thought to stimulate the self-renewal of cancer stem cells [31]. The primary therapeutic option targeting VEGFR is to use tyrosine kinase inhibitors (TKIs). Sunitinib, the most widely used TKI, yielded encouraging outcomes in response and survival when compared with interferon alpha (IFN-α) and thus became the new standard of care for advanced ccRCC [1,32]. Other common TKI monotherapy alternatives to sunitinib include cabozantinib and pazopanib [1]. 

ICIs demolish tumors by taking the ‘brakes’ off the immune system. In the tumor microenvironment (TME), the upregulation of immune checkpoint proteins gives rise to the exhaustion of CD4+ and CD8+ T cells and prevents the apoptosis of regulatory T cells (Tregs) [33]. The FDA has approved several ICIs, including PD-1 receptor inhibitors (cemiplimab, nivolumab, and pembrolizumab), PD-L1 inhibitors (atezolizumab, avelumab, and durvalumab), and CTLA-4 inhibitors (ipilimumab), for use in all patients. Depending on the patients’ medical conditions, dual ICI treatments with an anti-PD-1 and an anti-CTLA-4, or an ICI in combination with a VEGFR TKI, are both standards of care for metastatic RCC patients [1]. 

MTOR inhibitors also serve as a standardized second-line therapy for mRCC after first-line treatment of VEGFR-TKIs [34]. By downregulating mTORC1 and its pathway, mTOR inhibitors prevent excessive anabolism and energy utilization in tumor cells. Moreover, recent studies show that an mTOR inhibitor combined with TKI provided a better objective response rate (ORR), disease control rate (DCR), and progression-free survival (PFS) than an mTOR inhibitor alone in second-line settings [34].

Cytoreductive nephrectomy (CN) has also become a standard of care for clear cell RCC after two randomized trials investigating the OS of patients receiving CN yielded positive results in 2001 [35,36]. However, the effects of CN remain a matter of much controversy. Studies and analyses show that patients with an International Metastatic RCC Database Consortium (IMDC) disease risk of ‘poor’ barely derive any benefits from CN [37]. In fact, CARMENA, a randomized phase III trial in patients with mRCC, demonstrated that sunitinib alone was not inferior to CN followed by sunitinib [38]. Still, patients who are not candidates for CARMENA, such as those with lower IMDC scores, are likely to profit from CN treatment [37].

## 3. General Physiological Role of the Adenosine A2A Receptor

Adenosine initiates its function with a variety of G-protein-coupled receptors (GPCRs). Adenosine receptors (AR or P1 receptors) have four subtypes: A1R, A2AR, A2BR, and A3R. The affinities of A1R, A2AR, and A3R for adenosine are high, whereas A2BR has a lower affinity [39]. A1R is coupled to the Gi/o protein and often has an inhibitory effect. A2AR is coupled to the aGs protein and results in a downstream AC/cAMP/PKA cascade when it binds. When the adenosine level is high, A2AR binding is favored, and its activation can lead to the downregulation of A1R.

A2AR is widely distributed in the central nervous system, immune system, and blood vessels. In the nervous system, adenosine plays a role as a neuroprotectant agent and regulates motor function. Therefore, adenosine receptors are widely recognized as therapeutic targets for treating neurological disorders. One such disease, Parkinson’s disease (PD), has FDA approval for A2AR antagonist treatment [40]. PD patients suffer from dopaminergic neuron degeneration in the midbrain. The dopamine pathways that connect the midbrain to the striatum control the basal ganglia; thus, any weakening of the dopaminergic neurons results in a relative activation of the basal ganglia indirect output pathway [41,42]. This activation eventually triggers PD symptoms. A2ARs are selectively distributed on the basal ganglia and the indirect output pathway. Moreover, adenosine seems able to promote the indirect pathway through A2ARs, which is why A2AR antagonists are considered a potential treatment for Parkinson’s disease [41]. Currently, the A2AR antagonist istradefylline serves as an effective adjunct to levodopa, the gold-standard PD therapy [43].

The use of A2AR antagonists has also attracted interest as an emerging treatment for Alzheimer’s disease (AD). Evidence suggests that the adenosine concentration in the cerebral cortex is higher in AD patients than in healthy people [44]. Furthermore, A2AR overexpression is linked to decreased cognitive performance and memory dysfunction, which are typical features of AD [41]. The level of A2A receptors in astrocytes, which are strongly involved in the pathological mechanism of AD, increases in AD patients [45]. In fact, the effect of caffeine therapy on AD partially results from the pharmacological blockade of A2AR [46].

In ischemic brain stroke, A2AR blockade also demonstrates a neuroprotective effect, mainly because A2AR mediates the release of excitatory amino acids, which play key roles in neuron death in cerebral ischemia [47]. Epilepsy is another disease that benefits from an A2AR blockade, as the pathological mechanism of epilepsy involves an A2AR-linked release of glutamate, one of the excitatory amino acids that can trigger epileptic discharges [41,48].

The activation of A2AR on both endothelial and smooth muscle cells can lead to coronary vasodilation and reactive hyperemia [49], as adenosine regulates coronary microvascular tone and coronary blood flow. In coronary artery diseases, the downregulation of A2AR decreases blood flow, increases the immune response, and promotes platelet aggregation, all of which enhance the risk of an acute syndrome [50].

Adenosine also activates the secretion of insulin via A2AR activity. The A2AR-mediated activation of the cAMP/PKA pathway promotes insulin release, thereby mimicking the development of hyperglycemia [51]. Adenosine is also neuroprotective against retinal disease, as A2AR agonists or antagonists can prevent neuroinflammation in several blinding diseases; however, the mechanisms still require deeper insights [52].

## 4. Physiological Role of Adenosine A2A Receptors in Tumors

**Figure 1:**
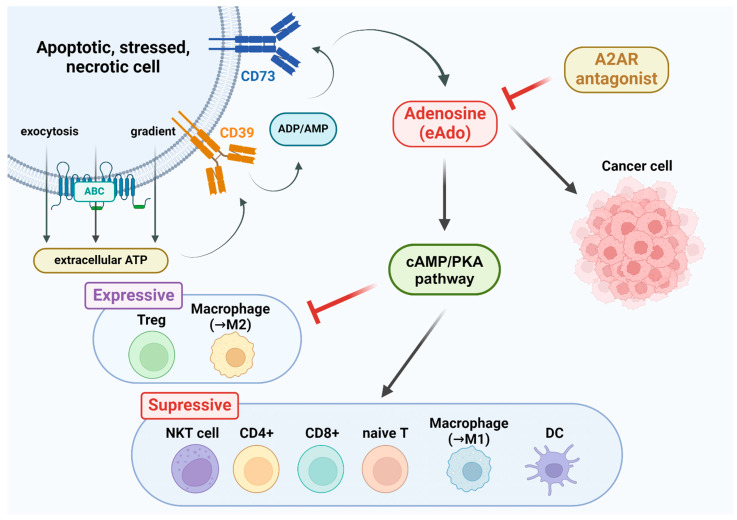
An illustration of the activation of adenosine A2AR and its influences on tumor growth. The occurrence of cell injuries, necrosis, or apoptosis triggers the release of extracellular ATP. CD39, a vascular ATP diphosphohydrolase, then converts the extracellular ATP into ADP/AMP, while CD37, which is similar to CD39, then converts ADP/AMP into extracellular adenosine (eADO). Excessive eADO stimulates the adenosine A2A and A2B receptors and activates the downstream cAMP/PKA pathway. This leads to the downregulation of the immune system. Immune cells that generate pro-inflammatory effects, such as dendritic cells (DCs), natural killer T (NKT) cells, and CD4+ and CD8+ T cells, are strongly suppressed. Conversely, immune cells that generate anti-inflammatory effects, such as regulatory T cells (Tregs), are induced. The ratio of macrophages that polarize into the M2 subtype instead of the M1 type also increases. All of these responses create an ideal microenvironment for tumor growth.

The activation of A2AR results in an enhancement of extracellular ATP. When hypoxia or tissue damage occurs, ATP is released from the intracellular space to the extracellular space. This leakage of ATP happens both passively and actively [53]. In stressed, apoptotic, or necrotic cells, ATP can be transmitted through chemical or electric gradients (Figure 1). It can also be released by exocytosis or via ATP-binding cassette (ABC) transporters, such as connexin hemichannels and pannexin 1 (PANX1) [54]. High levels of ATP can serve as damage-associated molecular patterns (DAMPs), which are endogenous factors that promote inflammatory responses and tumor progression [55]. Extracellular ATP is degraded into adenosine by exoenzymes CD39 and CD73, with CD39 hydrolyzing ATP to ADP and AMP and CD73 hydrolyzing AMP to adenosine. The overexpression of CD39 and CD73 can be observed in the tumor immune microenvironment (TIME) and in several types of immune cells. Moreover, these exoenzymes are recognized as novel checkpoint inhibitors, and the expression of CD73 is linked to multiple tumor signaling pathways [56,57].

Excessive extracellular adenosine (eADO) interacts with anti-inflammatory A2A and A2B receptors. The upregulation of adenosine receptors is followed by the activation of the downstream cAMP/PKA pathway. This limits the duration and extension of inflammation and affects immune cells in various ways [58].

The activation of A2AR suppresses anti-tumor immune responses. Among the four adenosine receptor subtypes, A2AR is most commonly expressed by immune cells. In the TME, the downstream signaling of A2AR suppresses the immune response of CD4+ and CD8+ T cells, as well as the proliferation of naïve T cells and the maturation of thymic T cells. It also downregulates the activation of NKT cells and decreases the amount of interferon gamma (IFN-γ) released by NKT cells [53]. Moreover, the activation of A2AR signaling triggers the generation of Treg cells, which mediate the expression of CD39/CD73 and inhibit the anti-tumor effects of the immune system. Macrophages respond to A2AR signaling by showing a tendency to polarize into the M2 subtype, which upregulates tumor proliferation and metastasis. Overall, in the TME, A2AR activation impacts immune cells in various ways that ultimately promote tumor proliferation (Figure 1) [53,59].

Adenosine also induces angiogenic responses in all four AR subtypes in the TME. As eADO increases, the stimulation of A2ARs and A2BRs activates cAMP signaling. The increase in cAMP can interfere with the differentiation of dendritic cells (DCs) and induce them to form myeloid DCs [57,60]. The myeloid DCs, in turn, facilitate VEGFR-mediated angiogenesis, thereby enhancing metastatic tumor growth [57]. The crucial role played by A2AR in promoting lymphangiogenesis and lymph node metastasis further proves its importance in tumor growth [61].

## 5. Types of Adenosine A2A Receptor Drugs

**Figure 2:**
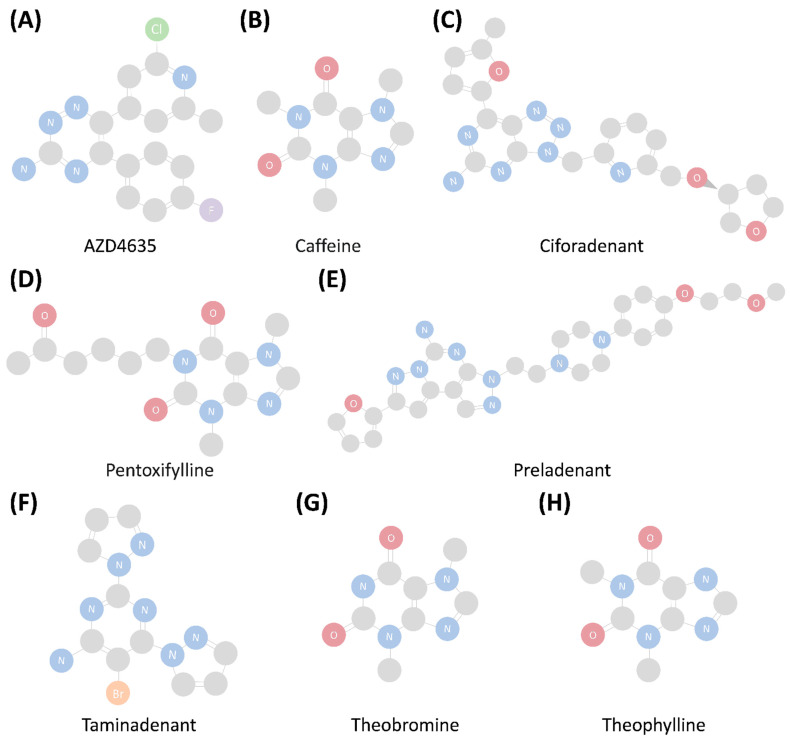
The adenosine A2A receptor drugs (**A**–**H**) that showed anti-cancer therapeutic effects in preclinical studies or clinical trials.

Currently, the FDA-approved drugs targeting the adenosine A2A receptor consist of agonists and antagonists/inhibitors. Several A2AR drugs have undergone cancer treatment research (Table 1, and Figure 2). Most of these drugs are A2AR antagonists, which can be classified as derivatives of xanthine, quinolone, and triazine.

Caffeine is one of the xanthine-derived antagonists. As a nonselective antagonist, caffeine has a similar affinity to all four adenosine receptors (Ki: 10.7 μM of A1R, 23.4 μM of A2AR, 33.8 μM of A2BR, and 13.3 μM of A3R) [62]. It is primarily recognized to have preventive and therapeutic effects on diseases of the central nervous system and cardiovascular system [63]. Much research also shows that high coffee consumption can decrease the risks of several types of cancer, although some studies have yielded contradictory results [64].

Caffeine has been suggested to prevent carcinogenesis in multiple ways. One of its effects is enhancing the anti-tumor immune response. The activation of the A2A receptor pathway is known to suppress the immunity of immune cells and the secretion of cytokines. In turn, the antagonism of A2AR induced by caffeine can promote the immune response. Other anti-tumor mechanisms of caffeine include the inhibition of cell cycle progression, the prevention of tumor angiogenesis, and the suppression of activated cell checkpoints in DNA-damaged cells.

Besides caffeine, other methylxanthines, such as theophylline and theobromine, are well known to have direct anti-tumor effects. Theophylline is a nonselective antagonist of adenosine receptors and acts as a bronchodilator or an anti-inflammatory drug in airway diseases [65]. Theobromine, by contrast, is used as a vasodilator, heart stimulant, and mild diuretic. Both theophylline and theobromine can prevent tumor progression and metastasis, possibly by repressing adenosine receptors and preventing angiogenesis in the TME [66,67].

Istradefylline (KW6002) is another xanthine-derived A2AR antagonist that mainly serves as a complementary drug for levodopa in Parkinson’s disease (PD). The connection between istradefylline and cancer has been poorly studied; however, recent research indicates that istradefylline can potentially reduce tumor growth. In the TME, this drug seems capable of decreasing malignancy-associated factors, increasing the production of pro-inflammatory cytokines, and inhibiting the tumor-inducing AKT/mTOR pathway [68,69].

Other A2AR antagonists associated with PD are preladenant and tozadenant. Preladenant has been shown to shorten the ‘off episode’ in PD (i.e., the period when the medication wears off and the symptoms recur) in a phase IIb trial. However, this therapeutic effect was absent in phase III trials [70]. Tozadenant also showed a tendency to shorten the ‘off episode’ of PD, but its further development was suspended when a serious adverse effect was reported in a phase III trial (NCT02453386). Fluorinated derivatives of preladenant and tozadenant are viewed as potential blockers of A2AR-activated immune inhibitory effects [71].

NIR178 (PBF509/taminadenant) is another A2AR antagonist under a series of investigations. In a mouse model and samples from patients with non-small cell lung cancer (NSCLC), NIR178 showed an ability to reduce lung metastasis and prevent the immune-evasive mechanisms mediated by A2AR [72]. In a phase I/Ib study, NIR178 administered as a single agent or combined with spartalizumab was well tolerated in patients with NSCLC [73].

Ciforadenant (CPI-444), an oral selective A2AR antagonist, has shown an ability to help restore T cell signaling and IL2 and IFNγ production in mouse tumor models [74]. It also demonstrated satisfactory therapeutic effects in a mouse tumor model when combined with anti-PD-L1 and anti-CTLA-4 drugs [75]. A study on a mouse model of combined ciforadenant and anti-PD-L1 therapy revealed that the T cells of nondraining lymph nodes showed decreased PD-1 expression, which lowered the threshold for effective anti-PD-1 therapy [74].

AZD4635 is an A2AR antagonist that can boost the function of T cells and enhance antigen presentation by CD103+ dendritic cells, as confirmed through in vitro assays of human and mouse model tumor cells. The combination therapy of AZD4635 and durvalumab, a PD-L1 inhibitor, has undergone phase I and phase II studies on prostate cancer patients. The phase I study increased the PFS in patients who received the combination therapy, but the phase II study did not yield similar results [76].

DZD2269 has also shown an ability to prevent A2AR-mediated immune inhibition in the TME. Its safety has been confirmed in a study of healthy participants, but another trial investigating its effects on prostate cancer patients has not yet reported its results [77].

Comparatively, agonists comprise only a small proportion of A2A receptor ADC drugs. Nevertheless, some of them have properties like those of A2AR antagonists. For instance, the A2AR agonist pentoxifylline is a xanthine derivative that is considered a therapeutic option for the prevention of tumor progression. Like theophylline and theobromine, pentoxifylline has an anti-angiogenesis effect, although the mechanism behind this effect is still unclear [78,79].

## 6. Clinical Trials Investigating Current Adenosine A2A Receptor Drugs in RCC

At present, several A2AR antagonists have undergone phase I/II clinical trials in RCC patients. A phase I/Ib clinical trial (NCT02655822) of ciforadenant, in combination with atezolizumab, a monoclonal antibody targeting PD-L1, was conducted on RCC patients. In that trial, approximately 72% of the patients were resistant or refractory to anti-PD-L1 treatments, but ciforadenant monotherapy showed efficacy, with a median PFS of 4.1 months and an OS of up to 69% at 16 months. The combination treatment exhibited even better results, including a median PFS of 5.8 months and a 90% OS at 25 months. The patients who responded to the ciforadenant monotherapy or combination treatment were positively associated with the expression of an adenosine-related gene [75,80]. Another clinical trial on the drug NCT05501054 is a phase I/Ib study investigating a combination of ciforadenant with ipilimumab and nivolumab, which are monoclonal antibodies against CTLA-4 and PD-L1, respectively. This study is estimated to be completed by November 2026.

Other less explored A2AR inhibitors that have also undergone phase I or II studies have included TT-10 (NCT04969315), an adenosine A2AR antagonist whose anti-tumor activity is evident in mouse models [81]. NIR178 (PBF509/taminadenant) is another example currently undergoing a phase I/Ib clinical trial (NCT04895748) in ccRCC patients as a potential adjuvant to DFF332, an HIF-2α inhibitor. HIF-2α is a downstream protein of the VHL gene, and its overexpression leads to VEGF activation [82]. NIR178 was also tested with the experimental medications NZV930 and PDR001 in a phase I/Ib study (NCT03549000) involving patients with NSCLC and multiple kinds of advanced malignancies, including RCC. NZV930 is a CD73-targeting drug, and PDR001 is an anti-PD-L1 medication. The prospect of immunotherapy and CD39/CD73/A2AR combination treatment has set high expectations for the clinical trial [59]. In the study report, the safety of the treatments was referred to as ‘acceptable,’ but little clinical benefit was observed, and no objective response occurred. Another study focusing on NIR178 is a phase II study (NCT03207867) featuring NIR178 and PDR001 combination therapy. This study recruited patients with solid tumors and diffused large B cell lymphoma (DLBCL).

Another phase I clinical trial (NCT03629756) is focused on etrumadenant (AB928), a selective A2AR and A2BR dual antagonist, in combination with zimberelimab (AB122), an anti-PD-L1 drug. This study is evaluating the responses of patients with advanced malignancies to the combination therapy. In this clinical trial, patients with RCC have shown no significant safety issues, although some inflammation and pulmonary edema have been observed.

## 7. Conclusions

Although the clinical indications of most A2AR drugs are cardiovascular and neurological diseases, increasing preclinical studies and clinical trials have already confirmed the feasibility of A2AR blockade as a new therapeutic strategy for cancer. So far, among all seven clinical trials investigating A2AR drugs in RCC, only two were completed, but one on ciforadenant yielded encouraging results (NCT02655822). Reportedly, more than 72% of the patients in this trial were resistant or refractory to anti-PD-L1 treatments. However, ciforadenant monotherapy’s anti-tumor activity was observed in these patients. Based on the PFS and OS, the efficacy of combined ciforadenant and Atezoizumab (a monoclonal antibody targeting PD-L1) treatment was even better than that of ciforadenant monotherapy. Moreover, the patients with high-level adenosine gene signature expression were significantly associated with tumor regression and showed a longer PFS after receiving treatment. Although there are still few studies on A2AR drugs in RCC, increasing numbers of clinical trials are emerging with the hope of creating new treatment options for refractory RCC patients.

## Figures and Tables

**Table 1:** Ongoing clinical trials investigating A2AR-targeting drugs in kidney cancer.

Drug	Trial	Phase	Number of Patients	Drugs Involved	Condition or Disease	Status
Ciforadenant (CPI-444)	NCT02655822	I/Ib	502	Atezolizumab	Renal cell cancer, metastatic castration-resistant prostate cancer	Completed
	NCT05501054	Ib/II	24	Ipilimumab, Nivolumab	Renal cell carcinoma	Recruiting
TT-10	NCT04969315	I/II	90		Renal cell cancer, castration-resistant prostate cancer, non-small cell lung cancer, head and neck squamous cell carcinoma	Active, not recruiting
NIR178 (PBF509/Taminadenant)	NCT04895748	I/Ib	40	DFF332, RAD001, PDR001	Renal cell carcinoma	Active, not recruiting
	NCT03549000	I/Ib	127	NZV930, PDR001	Renal cell carcinoma, non-small cell lung cancer, triple-negative breast cancer, pancreatic ductal adenocarcinoma, colorectal cancer microsatellite stable, ovarian cancer, metastatic castration-resistant prostate cancer	Terminated
	NCT03207867	II	315	PDR001	Renal cell cancer, non-small cell lung cancer, pancreatic cancer, urothelial cancer, head and neck cancer, diffused large B cell lymphoma, microsatellite stable colon cancer, triple-negative breast cancer, melanoma, metastatic castration-resistant prostate cancer	Terminated
Etrumadenant (AB928)	NCT03629756	I	48	zimberelimab (AB122)	Renal cell carcinoma, non-small cell lung cancer, squamous cell carcinoma of the head and neck, breast cancer, colorectal cancer, melanoma, bladder cancer, ovarian cancer, endometrial cancer, Merkel cell carcinoma, gastroesophageal cancer, castration-resistant prostate cancer	Completed

## Data Availability

All analyzed data are included in this article. Additional information is available upon request.
